# Median nerve conduction studies in rabbits

**DOI:** 10.1186/s12868-020-00584-2

**Published:** 2020-08-17

**Authors:** Basak Mansiz-Kaplan, Secil Pervane-Vural, Koray Gursoy, Baris Nacir

**Affiliations:** 1 Department of Physical Medicine and Rehabilitation, Ankara Training and Research Hospital, University of Health Sciences, Ankara, Turkey; 2 Reconstructive, and Aesthetic Surgery, Department of Plastic, Ankara Training and Research Hospital, University of Health Sciences, Ankara, Turkey

**Keywords:** Rabbit, Nerve conduction studies, Compound muscle action potential amplitude, Distal latency, Median nerve

## Abstract

**Background:**

When planning nerve conduction studies using animal models, the sciatic nerve is often used and the upper extremity nerves are not preferred due to the size of laboratory animals. This study aimed to present the method and mean values of median nerve conduction studies in laboratory rabbits. Fifty-five six-month-old male New Zealand white rabbits weighing 2 to 2.5 kg were included in nerve conduction studies performed under anesthesia. The compound muscle action potential amplitude and distal latency values were recorded for the median motor nerve with the electrodes placed on the flexor digitorum superficialis muscle and tendon.

**Results:**

A total of 110 median nerves were evaluated. The mean amplitude of the median nerve was 30.6 ± 6.8, mV the median nerve distal latency was 1.3 ± 0.2 ms, and the mean intensity of stimulation inducing a response was 2.5 ± 1 mA.

**Conclusions:**

The mean values obtained by the median motor nerve conduction method in this study can act as a guide for future nerve interventions undertaken in the upper extremities.

## Background

A nerve conduction study is a valuable laboratory method for assessing nerves. A standard measurement method is available for human beings with the data for each nerve having been standardized. In recent years, nerve intervention studies on laboratory animals have increased. However, there is no standardization for these subjects and reference ranges have not been determined. The sciatic nerve, the largest nerve of the mammals’ body, is widely used in studies conducted with rats and mice, and the median nerve is not preferred due to its smaller size.

Median nerve injuries are common peripheral nerve injuries in humans. The most common cause of median nerve injury is wrist lacerations in young patients and motorcycle accidents at later ages [1]. Carpal tunnel syndrome, characterized by entrapment of the median nerve at the wrist level, is the most frequent entrapment neuropathy [2]. Due to this high prevalence of both nerve injury and entrapment, researchers often conduct studies with laboratory animals to offer a better understanding of the median nerve. For intervention studies related to the median nerve, rabbits are preferred due to their size. Intervention studies related to the median nerve of rabbits are available in the literature, albeit being limited in number. To investigate the median nerve, the thenar muscles are usually utilized [3–11]. To date, the flexor digitorum longus muscle has only been used in one study [12]. Furthermore, in the literature, there is a lack of methods regarding nerve conduction studies and data regarding the mean conduction values for the median nerve. This study aimed to describe a method for the motor conduction studies of the median nerve and present the mean nerve conduction values obtained from the flexor digitorum longus muscle in rabbits.

## Results

A total of 110 median nerves belonging to 55 rabbits were evaluated. Table 1 presents the results of the mean compound muscle action potential amplitude (30.6 ± 6.8 mV)), distal latency (1.3 ± 0.2 ms) and mean intensity of stimulation (2.5 ± 1 mA) inducing a response for the median nerve. When the median motor nerve conduction studies of the right and left extremities were compared, no statistically significant difference was found between the two sides (Table 2).

**Table 1:** The mean values of median motor nerve conduction studies

	Mean ± SD	Min–Max
Amplitude (mV)	30.6 ± 6.8	16.7–44.5
Distal Latency (ms)	1.3 ± 0.2	1.02–1.86
Intensity of stimulation (mA)	2.5 ± 1	1–6

*SD* standard deviation

**Table 2:** The mean values and comparison of the results of the right and left median nerve conduction studies

	Extremity	Mean ± SD	P value
Amplitude (mV)	R	30.3 ± 6.9	0.62
	L	30.9 ± 6.7	
Latency (ms)	R	1.3 ± 0.1	0.43
	L	1.3 ± 0.1	
Stimulation (mA)	R	2.5 ± 1	0.83
	L	2.5 ± 1.1	

*SD* standard deviation

## Discussion

In this paper, we share the method for the conduction studies of an important nerve of the upper extremity, the median nerve, in rabbits and the mean values we obtained using this method.

In this study, we chose a method that is less frequently adopted in the literature; i.e., we recorded the conduction values in the flexor digitorum superficialis muscle, similar to a previous report by Baoguo et al. [12]. The flexor digitorum superficialis muscle has more volume than the thenar muscles. Therefore, we thought that more objective data could be obtained recording from flexor digitorum superficialis muscle, in the changes to be observed as a unit in intervention studies. On the other hand, as the studied area becomes smaller, it may necessary to use more specific and expensive electrodes in nerve conduction studies with animals. For all these reasons, we preferred the less studied method using flexor digitorum superficialis muscle. Baoguo et al. only calculated the velocity of the median motor nerve after performing elongation. Since they did not provide any data concerning nerve conduction prior to intervention, we were not able to compare our values; however, they provided a description of the method they used, in which they applied stimulation at 5 mm and 40 mm proximal to the nerve [12]. In the current study, when we attempted to stimulate the nerve at the 5 mm proximal, but did not attain any response. This may be due to the size of our stimulator. We achieved the best response from 30 mm proximal to the recording electrode, which corresponds to the point where the median nerve becomes superficial. We had difficulty isolating the median nerve response due to the closeness to the brachial plexus resulting from a more proximal stimulation to calculate the velocity. Therefore, we do not have the velocity data for the median motor nerve.

In other studies in the literature, the recording electrodes were placed in the thenar muscles and needle electrodes were preferred [3–12]. In this study, we first attempted to undertake nerve conduction studies using surface electrodes before needle electrodes based on the idea that it would provide more accurate reference values. However, due to the small size of the area studied, the surface electrode method was not successful. The study was completed with needle recording electrodes similar to the procedures in the literature and as reported by other researchers [3–8], we used 30 mm proximal stimulation. The reason for our preference of 30 mm was that at this point, the median nerve was superficial enough to be seen with the naked eye, as explained above.

For comparison purposes, we only found three studies in the literature that provided data on nerve conduction before interventions [4–6]. However, these studies used the thenar muscles; i.e., their method was different. Only one study used the flexor digitorum longus muscle, but a comparison was, again, not possible since the authors did not share the mean data [12]. Vanhees et al. conducted a study with 50 female rabbits (unspecified breed) weighing 3.6 to 6.2 kg, reporting the median motor amplitude as 2.5 mV and latency as 1.5 ms [4]. Investigating 42 female New Zealand rabbits weighing 3.6 to 6.2 kg, Moriya et al. presented the mean amplitude value as 2 to 2.5 mV [5]. In another study, 15 male New Zealand rabbits with a weight of 4.0–4.5 kg were evaluated. The median motor nerve latency values before the procedure were provided and the mean latency was measured as 1.5 ms [6]. In the current study, the median motor nerve latency was slightly shorter (1.3 ± 0.2) compared to the literature. This can be attributed to the measurement performed more proximally than the other studies. However, our median nerve amplitude values were higher than reported in the literature probably due to our weaker and younger sample, the differences in the method used, and the larger sample size.

In this study, different from the literature, we also recorded the mean intensity of stimulation that induced a response and we consider that the related results will guide future studies. Based on our findings, we recommend not exceeding 6 mA as the maximum stimulation intensity since the values above this level may result in the stimulation of neighboring nerves in animals of this size.

When the median motor nerve data of both extremities were compared, no significant difference was found in the latency, amplitude and stimulus intensity values. Therefore, we think that the use of the right or left median nerve in intervention studies will not lead to any difference in the results.

Our study had certain limitations. First, we had to use needle electrodes as the recording electrode. If we had been able to perform measurements with surface electrodes, we could have presented data of reference value; however, as explained above, we were not successful with our first attempt using surface electrodes due to the small size of the area studied. Similar to our study, in the literature, needle electrodes are used as the recording electrode. Another limitation was that we were not able to calculate the median motor velocity, which can probably be linked to the same reason; i.e., the examined area being small. However, we believe that the latency and amplitude values we obtained for the median motor nerve are sufficient for planned intervention studies.

## Conclusion

It is known that the small size of laboratory animals makes it difficult to perform interventions on the upper extremity nerves. We consider that the method used and the mean data obtained from this study will guide future median motor nerve studies using animal models.

## Methods

After obtaining approval from the local animal subjects ethics committee of our hospital, 55 six-month-old male New Zealand white rabbits weighing 2 to 2.5 kg were included in the study. The rabbits were acquired from a private source which’s name is “Saki Yenilli Deney Hayvanları Üretim ve Uygulama Laboratuvarı”.

### Preparation

All the rabbits were anesthetized before the median nerve conduction study. A cocktail containing ketamine (1.5 ml, 100 mg/ml), acepromazine (0.5 ml, 10 mg/ml), and xylazine (0.5 ml, 20 mg/ml) was prepared. The cocktail was administered intramuscularly to each rabbit at 0.5 ml/kg. The ventral aspect of the upper extremity of each rabbit was shaved prior to the nerve conduction studies.

### Nerve conduction studies

**Fig. 1:**
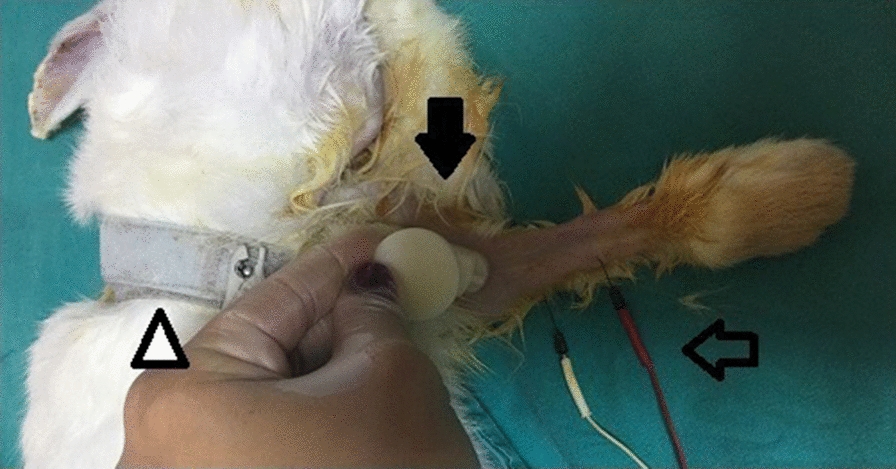
Placement of nerve conduction electrodes in the rabbit. Triangle: ground electrode, filled arrow head: stimulator, unfilled arrow head: recording electrodes

**Fig. 2:**
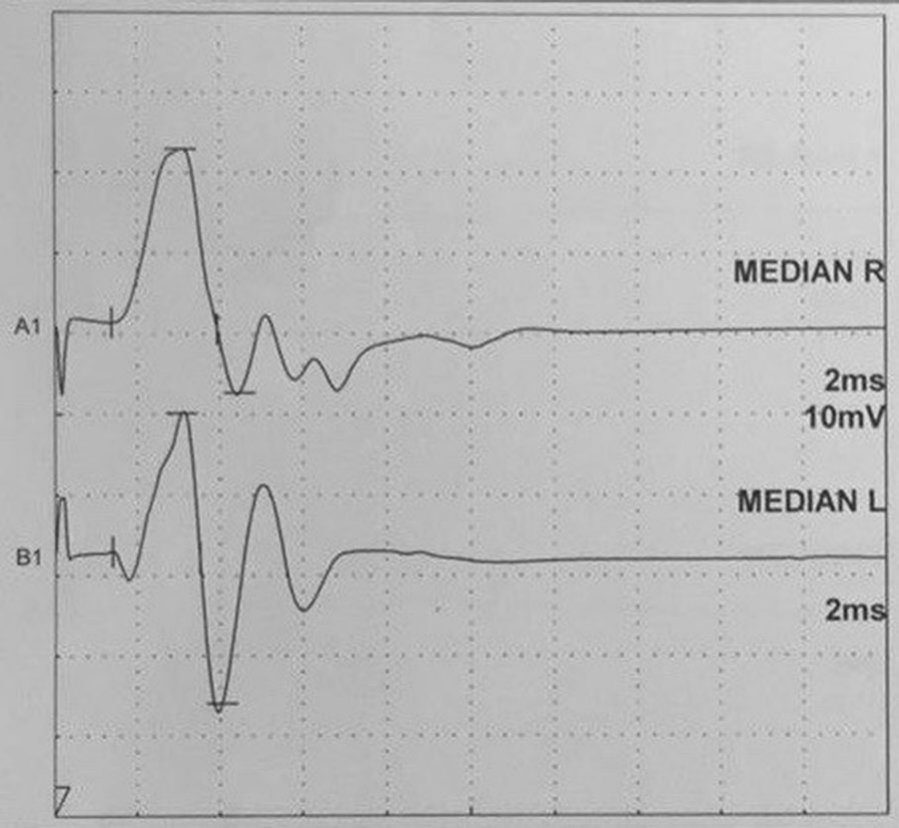
The response recorded from the flexor digitorum superficialis muscle for the median motor nerve

All median nerve conduction studies were performed using a Nihon-Kohden Neuropack M1 (Tokyo, Japan) device. Room temperature was maintained at 25 °C. The extremity temperature was measured with a digital needle thermometer and maintained at 34–36 °C. The technical specifications of device are listed: stimulation rate is 1 Hz, sampling time is 100 µs and filter settings are 5 kHz for hi-cut, 10 kHz for low-cut. The monopolar needle recording electrodes were positioned as follows: the anode electrode in the middle of the flexor digitorum superficialis muscle, the cathode electrode on the tendon, and the ground electrode on the back of the rabbit. The bipolar stimulator was placed 30 mm proximal to recording anode electrode, where the median nerve becomes superficial (Fig. 1). Both the anode and cathode were placed over the median nerve. The distance between anode and cathode polar of stimulator was 2 cm and the anode polar was positioned distally. The stimulation intensity was gradually increased until the supramaximal motor response was achieved for the median motor nerve. The compound muscle action potential amplitude, distal latency and mean intensity of stimulation for the median nerve were recorded for the median motor nerve. Distal latency was measured from the beginning of the stimulus artifact to the onset of the action potential and the compound muscle action potential amplitude was measured peak to peak. The study was conducted bilaterally. The response recorded from the flexor digitorum superficialis muscle for the median motor nerve was shown in Fig. 2.

The rabbits were not euthanised after the study.

### Statistical analysis

The Statistical Package for the Social Science Program (SPSS version 15.0, IBM, Armonk, NY, USA) was used for statistical analysis. Descriptive statistics were applied for the mean, minimum and maximum values. The independent sample t-test was used to compare the data between the right and left extremities.

## Data Availability

The datasets are available from the corresponding author on reasonable request.
